# Can HIV vaccines be shared fairly? Perspectives from Tanzania

**DOI:** 10.1186/s12910-022-00874-w

**Published:** 2022-12-15

**Authors:** Godwin Pancras, Mangi Ezekiel, David Nderitu, Erasto Mbugi, Jon F. Merz

**Affiliations:** 1 Department of Bioethics and Health Professionalism, Muhimbili University of Health and Allied Sciences, P.O. Box 65001, Dar es Salaam, Tanzania; 2 Department of Behavioral Sciences, Muhimbili University of Health and Allied Sciences, P.O. Box 65001, Dar es Salaam, Tanzania; 3 Department of Philosophy, History and Religious Studies, Egerton University, P.O. Box 536-20115, Egerton, Kenya; 4 Department of Biochemistry, Muhimbili University of Health and Allied Sciences, P.O. Box 65001, Dar es Salaam, Tanzania; 5 Department of Medical Ethics and Health Policy, Perelman School of Medicine, University of Pennsylvania, Blockley Hall Floor 14, 423 Guardian Drive, Philadelphia, PA 19104-4884 USA

**Keywords:** HIV vaccines, Benefit-sharing, Distributive justice, Global south, Tanzania

## Abstract

**Background:**

For over 35 years, Africa has continued to host HIV vaccine trials geared towards overturning the HIV/AIDs pandemic in the continent. However, the methods of sharing the vaccines, when available remain less certain. Therefore, the study aims to explore stakeholders’ perspectives in the global South, in this case, Tanzania, on how HIV vaccines ought to be fairly shared.

**Methods:**

The study deployed a qualitative case study design. Data were collected through in-depth interviews and focus group discussions with a total of 37 purposively selected participants. This included researchers, institutional review board members, a policymaker, HIV/AIDS advocates, and community advisory board members. The data obtained were inductively and deductively analyzed.

**Results:**

Findings indicate that HIV vaccines can be shared fairly under the principles of distributive justice (contribution, need and equality). Thus, contribution-based sharing ought to be utilized upon the necessity to prioritize vaccine access or subsidized trial benefits to host communities. Need-based sharing ought to be considered for non-host communities that are at an increased risk of HIV infection. Lastly, equal-based sharing would be useful at later stages of vaccine distribution or when the aforementioned principles are deemed morally inappropriate. However, none of the benefit-sharing approaches is free of limitations and a counterbalancing sense of unfairness.

**Conclusion:**

Fair sharing of HIV vaccines, when available, ought to be informed by the contribution, need and equality principles of distributive justice. Countries in the global south including Tanzania are likely to be prioritized during the distribution of the HIV vaccines due to their participation in HIV vaccine trials and due to the disproportionate HIV burden evident in the region.

## Introduction

**S**ince the earliest trials in Zaire (now the Democratic Republic of Congo), the African continent has been home to HIV vaccine trials for over 35 years [1]. At the same time, the continent is still home to over 50% of all the new cases of HIV infection in the world [2]. The United Republic of Tanzania (hereby referred to as Tanzania), is among the few African countries that have availed their population to participate in HIV vaccine development trials [3]. Most of these trials have been limited to phases 1 and 2 recruiting healthy volunteers [4]. The country records over 7000 new HIV cases annually with nearly 5% of its adult population testing HIV positive [5]. This could explain why the country has already hosted about eight HIV vaccine trials to date [4].

The vaccine is a public good, but it remains challenging how it could be shared when it becomes available. International research guidelines emphasize making available identifiable benefits but then fall short of addressing the basis on which such benefits ought to be shared [6–9]. This shortfall is echoed by the Tanzanian research guidelines [10]. The Joint United Nations Programme on HIV/AIDS (UNAIDS) and World HealthOrganization (WHO) call for a prior ‘agreed plan’ between researchers, sponsors and other key stakeholders [11]. But if the basis for such agreements are ethically unguided the power differential that might exist risk attracting ‘agreed’ inequalities. Disagreements between parties further complicate the need to share the trial benefits [12]. According to Dauda et al. benefit sharing is an “act of giving something in return to the participants, communities, and the country that have participated in global health research or bioprospecting activities” [13].

In this study, we conceptualize benefits as viable and already licensed HIV vaccines for regular use by the WHO. As part of the contribution towards addressing this challenge, our study empirically utilizes the principle of distributive justice. According to Kaufman, the distributive justice principle “is concerned with the fair distribution of the burdens and benefits of social cooperation among diverse persons with competing needs and claims” [14]. Unlike other principles of justice, the distributive justice principle derives its strength from a due consideration of benefits in research conducted within communities with limited resources [13]. Moreover, the principle claims equality, contribution and need to be important considerations towards attaining fairness in benefit-sharing practices [15]. Thus, the principles of equality call for an equal share of benefits between parties; contribution assigns benefits based on the merit of the parties involved; and need calls for benefits to be assigned to the worse-off even if they had not participated in developing a vaccine [15, 16]. Therefore, this study aimed to explore the basis for sharing vaccines that would result from HIV vaccine trials conducted in resource-limited settings like Tanzania.

## Methods

Data was collected as part of an ongoing study of the ethical implications of sharing HIV vaccine trial benefits in Tanzania. The study employed a qualitative case study design informed by the constructivism inquiry. The design facilitated the exploration of the phenomenon holistically and in a real-life context from diverse perspectives [17]. The study was conducted in two regions in Tanzania: Mbeya which is predominantly semi-urban and Dar es Salaam which is predominantly urban. The two regions have hosted almost all HIV vaccine trials conducted in the country. Participants of the study were selected purposively and included: experienced researchers involved in HIV vaccine trials, Institutional Review Board (IRB) members who had participated in reviewing HIV clinical trials; Community Advisory Board (CAB) members who had represented their communities in HIV vaccine trials; a policymaker; and members from HIV advocacy groups. To be included in the study all participants had to be 18 years of age and above. Information from participants was collected using face-to-face IDIs and FGDs except two participants who offered their response in writing. However, a detailed description of the methods employed in this study has been fully explained elsewhere [18].

In this study, two categories of respondents were added including individuals from HIV/AIDS advocacy groups and one policymaker as previously noted. Most HIV/AIDs advocates were approached through the National Council of People living with HIV/AIDS (NACOPHA) in Mbeya and one from the European and Developing Countries Clinical Trials Partnership (EDCTP) which is a research funding organization. During interviews, participants were asked specifically how they think HIV vaccines could be shared then followed by probes on the three principles of distributive justice: contribution, need and equality.

Ethical considerations.

The study was approved by the Research and Publication Committee of Muhimbili University of Health and Allied Sciences (MUHAS-REC-4-2019-03.E2) and the National Health Research Ethics Committee (NatHREC*)* (NIMR/HQ/R.8a/Vol. IX/3543). All study participants voluntarily provided individual written consent. During interview sessions, focus group participants were asked to identify one another by their given numbers and not by their names.

## Results

### Description of study participants

The study included a total of 37 participants aged between 24 and 80 years (Table 1). Part of the findings in this study have been documented in our previous study which included a total of 29 participants [13]. Our prior study examined what constitutes benefits in HIV vaccine trials, drawing on interviews with 29 key stakeholders. Here, we narrow our inquiry to a specific benefit [HIV vaccines] identified by our subjects, focusing on how HIV vaccines, if and when they become available, ought to be made available in Tanzania. This analysis draws from those prior interviews, as well as data from 8 interviews (7 HIV/AIDs advocates and 1 policy maker) as described in Table 1.

**Table 1:** Social demographic characteristics of study participants

Description	IDI (n = 22)	FGD (n = 15)
*Gender*		
Female	9	5
Male	13	10
Age (years): mean (SD)	54 (12.7)	48 (12.7)
*Education status*		
Primary	2	2
Secondary	2	6
Diploma	–	5
University	18	2
*Employment status*		
Employed	17	6
Self-employed	5	9
Researchers’ experience in years (mean)	20	–
IRB working experience in years (mean)	12	–
CAB membership period in years (mean)	–	7

### Description of findings

Our qualitative analysis yielded one overarching theme, 3 main themes, and 7 sub-themes, as shown in Table 2. These themes, with representative examples drawn from interviews and the focus group, are described below.

**Table 2:** Summary table of findings

Overarching theme	Theme	Sub-theme
*The distributive justice principles ensure fairness in sharing HIV vaccines*	Contribution-based sharing of HIV vaccine trial benefits	Reciprocity to participants and host countries
		Limitation to contribution-based sharing
	Need-based sharing of HIV vaccine trial benefits	Stopping the HIV epidemic
		Limitation to need-based sharing
	Equality-based sharing of HIV vaccine trial benefits	All at risk for HIV infection
		Inclusivity in sharing HIV Vaccines
		Limitation to equal-based sharing

#### Contribution-based sharing of HIV vaccine trial benefits

Regarding the question of how the benefits of HIV vaccine trial ought to be shared, respondents reported that it was right for those who participated in HIV vaccine trials to be prioritized **including their communities** and countries when the vaccine becomes approved:

“…those countries whose governments agreed and participated in the efforts to solve the problem should then be the first to benefit…” (FGD 1: CAB #01)

With further analysis of data, the following sub-themes were identified.

##### Reciprocity to participants and host countries

To study participants, prioritizing trial participants to receive the vaccine seemed reasonable since it is a show of appreciation for risks incurred and contributions made. It was believed that, unlike others, trial participants carry the burdens of participating in HIV vaccine trials in terms of risk-taking, time, pain from trial procedures, and inconveniences. Apart from individuals and communities, consideration for prioritization was also extended to include countries hosting the trials. One study participant stressed that:

“…if the country has spent resources and costs in participating in the trial it is best to start benefiting first.” (IDI: HIV advocate #1).

Resource-limited settings could not financially support HIV vaccine trials but making their population available for trials was equally considered a contribution. One researcher noted that:

“…when dealing with resource-poor settings whose contribution may not be as much in terms of financial contribution to the research activities but they contribute indirectly by availing their populations to the conduct of trials” (IDI: Researcher #2)

Furthermore, it was noted the informed consent process delineated how and whether or not participants would benefit as noted below during one of the FGDs:

“…we believe that those who entered the trial were told all the procedures and how they would benefit and how they might not benefit.” (FGD 2: CAB #4).

To ensure that HIV vaccines are made available to host communities, one principal investigator reported communicating about this matter with some pharmaceutical companies to try to secure assurances that the vaccine would be accessible to the population if and when it becomes available:

“…we tried as much as possible to entice the vaccine manufacturer to make some kind of a commitment that should this prove to be efficacious then a possibility should be made for the population in resource-poor settings to get the vaccine at subsidized price…but the manufacturers will not make a one hundred per cent commitment.” (IDI: Researcher #2)

##### Limitation to contribution-based sharing

Some study participants were skeptical and raised concerns about prioritizing trial participants or host countries/ communities to receive HIV vaccines over the ones that did not host or participate in the clinical trials. The four main reasons offered were altruism, nationalism, and difficulties in weighing contributions and tracing the potential beneficiaries. For altruism, study participants believed that people did not participate in HIV vaccine trials for their own sake. Those who had an opportunity to be included, they argued, should count themselves as heroes and heroines acting to save the community or country from the HIV epidemic. Benefits originating from their participation are to be shared with the whole community. One of the study participants who happened to have participated in an HIV vaccine trial stated:

“I volunteered to participate in the HIV vaccine trial for the benefit of the whole community. So it is only right that these vaccines should be provided…to all participating and non-participating communities…” (FGD3: CAB #4)

Additionally, it was observed that the concept of prioritization based on contribution was raised more often when study participants compared host and non-host countries than when comparing groups/communities within the same country. It was emphasized that participating communities within the same country did so to benefit fellow countrymen and women. One of the researchers noted that:

“…in case the vaccine becomes available consideration will be given to a country that has put a lot of effort into its acquisition and then others will follow suit…” (IDI: Researcher #6)

How to weigh contributions as a basis for assigning HIV vaccines was seen as a challenge because different parties have contributed differently. Consequently, one of the study participants saw it as a challenge, regarding the challenges of tracing and identifying specific HIV vaccine trial participants to be allotted the vaccine that they helped develop:

“…I don’t see how they will find the [HIV vaccine] trial participants and some of them may have died already, take into consideration that HIV was killing very fast in the past years.” (IDI: IRB#2)

#### Need-based sharing of HIV vaccine trial benefits

The theme was identified after asking study participants how they thought HIV vaccines should be shared when they become available. Study participants mentioned need and risks being the key consideration in the distribution of the vaccines. It was assumed that if people in need (those who are at elevated risk of infection) are not given priority in accessing vaccines it would be impossible to curb the epidemic. Stopping the HIV epidemic and the limitations to need-based sharing of HIV vaccines were identified as sub-themes.

##### Stopping the HIV epidemic

Study participants opined that prioritizing people in need of the HIV vaccine would stop the HIV epidemic. They defined those in need of the vaccine both population and country-wise. With population-wise categorization, terms like ‘at risk’ populations and ‘vulnerable’ populations were used, accompanied by examples such as female sex workers, youth and people injecting drugs to mention a few. Other groups cited were healthcare workers and long-distance truck drivers. For country-wise categorization, study participants did not only require countries with a higher prevalence of HIV to be given priority but also countries that failed to achieve the 90-90-90 target proposed by the Joint United Nations Programme on HIV/AIDS (UNAIDS) [14]:

“…when you reach the 90-90-90 target it shows the success… those who are below the 90-90-90 target they should be the one to be given priority” (IDI: Reasearcher#3)

##### Limitation to need-based sharing

Participants were worried about the sharing of HIV vaccines based on need because of the difficulties in identifying and prioritizing people who might most need the vaccine. One study participant reported that:

“…everybody will tell you he has a need but we have to scrutinize and say this is a real need at this point…” (IDI: Researcher #3)

Moreover, during interviews participants perceived that the need for an HIV vaccine was associated with being at ‘risk’ for HIV infection. Thus female sex workers, youth and other vulnerable populations have a greater need for an HIV vaccine because they are at heightened risk of acquiring HIV compared to others.

“If there is a limited amount of [HIV] vaccine to be shared, the priority should be to young girls who are sexually active, sex workers, intravenous drug users and other groups at risk.“ (IDI: Researcher #4)

#### Equality-based sharing of HIV vaccine trial benefits

Contrary to the first and the second theme, participants wished that when the HIV vaccine becomeavailable it should be allotted to everyone regardless of his or her contribution to the trial or need. By further analysis of data, three sub-themes were identified: we are all at risk for HIV infection, inclusivity and limitations to equal sharing of HIV vaccines.

##### All at risk for HIV infection

Study participants believed that everyone on the planet in one way or another is at risk of acquiring or being affected by HIV because the world has become a village. Thus, some participants thought that everyone should be able to access the vaccine when it becomes available. During interviews, they further noted that pointing out certain populations as the ones that bear increased risk of HIV, is in their views, a misunderstandingof how the virus is transmitted within the community. For example, female sex workers were repeatedly cited by study participants as being at risk for HIV, while their customers, including married couples, youth and other people in the community, are regarded as low-risk populations.

“…this woman who is in the sex trade is not going to do business with only men who pay for sex but also with other ordinary people…” (IDI: HIV advocate #4)

##### Inclusivity in sharing HIV vaccines

Some study participants pointed out that access to the HIV vaccine was a right deserving similar weight to other human rights. Thus allocating the vaccine to people or communities that participated or offered resources which led to the vaccine discovery would risk bias amongst social classes, creating divisions within the community. One way to avoid this is to share the vaccine with all. One study participant recommended that:

“…we should not deny any of those societies the benefits of vaccine trial simply because they have no resources to support clinical trials” (IDI: Researcher #2).

In addition, some study participants noted that some potential host communities did not elect to be included in any HIV vaccine trials, so they thought it would be unreasonable to prioritize access in communities simply because they participated in a trial. One CAB member illustrated this by providing an example:

…when you say that Mbeya [Tanzania administrative region] should get the vaccines first, the people of Mwanza [Tanzania administrative region] will also say why not us...did you tell us to participate and we refused? Therefore, the [HIV] vaccines should be for all… (FGD 3: CAB #1)

##### Limitation to equality-based sharing

The belief that an HIV vaccine should be shared equally was opposed by some of the study participants. First, they expressed the view that it is unrealistic to fulfil such a goal due to the logistical challenges of mass production and distribution to ensure everyone gets an HIV vaccine. Second, some interviewed participants found it unreasonable for those who contributed greatly to the vaccine discovery to scramble or be considered equal to others who did not take part. One of the researchers noted that:

“Everyone should have an opportunity [to access HIV vaccine] if resources will allow but I think there are people who should be given priority… I don’t think even if the vaccine is available tomorrow we will have the resources to procure 40 million doses.” (IDI: Researcher #3)

An IRB member added that:

“…there is no equal sharing, you will give benefits to your people first and when your people are covered you can think of others (IDI: IRB#2).

## Discussion

HIV vaccines can be shared fairly if the parties involved are ethically informed by the underlying considerations and limitations of contribution, need and equality benefit-sharing mechanisms. This would mean that countries like Tanzania have a moral claim to first access to HIV vaccines due to both: participation in clinical trials and the high HIV burden.

Morally, it could be argued that goods or benefits earned as a result of cooperation ought to be shared among cooperating parties [16]. Consequently, the same argument could be used to claim for benefits of HIV vaccine trials conducted in developing countries like Tanzania. Moreover, this fulfils the requirements of the principle of reciprocity which emphasizes ‘what people deserve as a function of what they have contributed to an enterprise or society’ [20]. In this study, reciprocity indicated the appreciation of trial participants’ contribution and risks incurred. An obligation to reciprocate benefits, in this case, the HIV vaccine, based on contribution and risks has attracted support from different scholars [21]. Similarly, the International Ethical Guidelines for Health-related Research Involving Humans explicitly state that “If the knowledge to be gained from the research is intended for use primarily for the benefit of populations other than those involved in the research…the research raises serious concerns about justice” [8]. The aforementioned propositionswould likely need to be grounded in some kind of agreed plan or contract to be morally justified as indicated in the UNAIDS/WHO guidance [11]. Interestingly, our study findings indicate that there is difficulty in obtaining assurance or commitment between responsible parties. For that cause, host communities remain less assured and blinded on whether they will ever be prioritized to receive the HIV vaccine when it becomes available. However, this should not be the question for resource-limited settings like Tanzania whose population has participated in HIV vaccine trials for nearly fifteen years. Thus, to uphold the principles of contribution-based benefit sharing it would necessitate researchers, sponsors and other key stakeholders to reciprocate the HIV vaccine as a benefit to trial participants and communities.

On the contrary, it was noted that it is impractical to allot HIV vaccines to individuals or communities simply because they had participated in a trial. Pointing out altruism, nationalism and challenges in weighing trial contributions as well as tracing the beneficiaries as the reasons. Thus, sharing benefits based on contribution stands to disregard altruism as an important element of research participation. People may participate in HIV vaccine trials because they feel obligated to do public good, with personal benefits being secondary [22]. Particularly in Tanzania, the altruistic need to lessen the HIV burden in the country has been established as a significant motive to participate in HIV vaccine trials [23, 24]. But again as a precaution, altruism is not absolute since some trial participants may exercise ‘conditional’ altruism: a consideration for self-benefits first before participating in the trial that would benefit others [25].

Moreover, in this study, the call to prioritize HIV vaccines emerged was more pronounced when comparing participating to non-participating countries than when comparing participating to non-participating communities within the same country. This notion of participation in research to benefit fellow countrymen or women risks inviting vaccine nationalism as a new reality in HIV vaccines as it is already being reported with vaccines for COVID-19 [26], avian influenza H5N1 and H1N1 [27] pandemics. Concerning difficulties in weighing contribution, like any other clinical trials, HIV vaccine trials involve different clinical phases and stakeholders. To ensure fairness one would need, first: weigh each party’s contribution to the vaccine development, second: rank significant contributions, with the highest contributors topping the list and vice versa: this is where the problem arises since no standardized tools or mechanism exist to weigh contributions and assign benefits resulting from a trial. Although international ethical guidelines emphasize the need to benefit participants and host communities but still attaining fairness would need a contribution weighing mechanism to be in place.

Again, topping the list does not necessarily indicate the need for the vaccine whatsoever. Unless need becomes a criterion which then could compromise the essence of the contribution-based sharing principles. In the meantime it is problematic how trial participants would be traced and if it is worth it because (1) vaccines take a long time from bench to bedside, for over 30 years of HIV vaccine trials, HIV vaccines remain elusive [28]; (2) the question of who should undertake such responsibilities including the logistics remains less certain. The CIOMS guideline had previously obliged researchers and sponsors to undertake such a responsibility [8]. As Haire and Jordens point out, researchers have a ‘direct duty of care’ to the trial participants [12]. Still, it might be easy to trace and prioritize participants from recent and late-phase clinical trials but the same could not be said for participants of the past early-phase trials.

To overcome the shortfalls of contribution-based sharing, sharing benefits according to need is crucial. For this study, the needy could be defined with reference to ‘at risk’ or ‘vulnerable’ individuals. Commercial sex workers, youth, long-distance truck drivers, and men who have sex with men among others are regarded as at-risk populations in need of the HIV vaccine. This is because almost 65% of new HIV infections occur in the aforementioned populations [29]. In Tanzania, HIV prevalence among female sex workers is three times higher compared to the general population [30]. From the study participants’ perspectives, a needy population or country could be defined based on HIV prevalence and the 90-90-90 target set by UNAIDS respectively. Thus, communities with high HIV prevalence are to be prioritized if the vaccine becomes available. The same for countries that have failed to reach the 90-90-90 target (that is, by 2020: 90% of all people living with HIV know their status, 90% of all people diagnosed with HIV to be on sustainable antiretroviral therapy and 90% of those on antiretroviral therapy have achieved viral suppression) [19]. Taking these two major factors into consideration, need-based sharing is warranted as a principle for sharing HIV vaccines. It is to be noted that the Sub-Saharan region, which hosts most HIV vaccine trials conducted in Africa, is home to two-thirds of people living with HIV globally. Most of these countries are still struggling to achieve the UNAIDS target [2, 4].

However, defining which sub-groups or populations need the vaccine could be challenging especially when there is blurred differentiation between the needy population and the at-risk population. That is, study participants assumed that the population at risk for HIV infection would eventually need an HIV vaccine when it becomes available. This assumption may not hold water in light of the evidence and current experience of COVID-19 vaccine hesitancy. Populations and regions around the world that are deemed to be disproportionately burdened by COVID-19 are reluctant and hesitant to be vaccinated including at-risk individuals like healthcare workers [31, 32]. Thus, there should always be a precaution if benefits are to be shared based on the need to avoid an assumptive causation relationship between risk and need.

Apart from contribution and need-based sharing, equality could also be proposed as an alternative to sharing HIV vaccines. The study participants believed that HIV vaccines should be shared with everyone because no one is immune from acquiring HIV and that includes populations presumed to be at low risk. Based on participants’ accounts, there is increasing sexual interaction between the general population and populations deriving the epidemic like commercial sex workers. Similarly, a meta-analysis study conducted by Hodgins et al. indicated that HIV prevalence was higher among men who ever paid for sex compared to those that did not [33]. Thus, the propagation of beliefs that priority to access vaccines should be to populations at increased risk could be seen as ignoring HIV transmission between the two groups which is fundamental to ending new HIV infections. But again, from the equity perspective, priority could be given to populations mostly deriving the epidemic than to the general population.

However, an appeal for an equal share of benefits could also avert a sense of bias and social classes among individuals, communities and regions. Again this has been noted with the distribution of COVID-19 vaccines in the global south whereby as of 26th February 2022 over half of the global population (71% of which reside in high-income countries) was fully vaccinated compared to 10.7% of the African population [34]. Participants found it unrealistic to equally share the HIV vaccines when available due to financial incapacity and logistical challenges. Most countries in the global south are already struggling to independently fund existing interventions against HIV [35]. This leaves the decision as to whether or not everyone gets the HIV vaccine to be made by international donors and organizations. That being the case, mechanisms should be established in advance not only to buy HIV vaccines but also to ensure their fair distribution in the global south.

The results of this study ought to be interpreted with consideration of its limitations and mitigations. The study was conducted qualitatively thus limiting the generalization of participants’ perspectives to other areas hosting similar or different vaccine trials. Moreover, the FGDs were conducted among CAB members in one region which could have limited the diversity of perspectives in this case study. However, these issues were mitigated by triangulation of the study areas (Dar es Salaam and Mbeya region), data collection methods (FGD and IDI) and including diverse groups of participants (researchers, CAB members, IRB members, a policymaker and HIV advocates) to explore the same phenomenon from multiple angles. Additional research is needed to inform the philosophical and normative approaches to sharing benefits of human research beyond HIV vaccines,considering the rise in North-South and South-South research collaborations.

## Conclusion

Sooner rather than later, the global community spearheaded by WHO would be obliged to ensure HIV vaccines (if and when licensed) are shared fairly. According to this study, the principles which ought to guide the sharing of HIV vaccines each possess reasonable strength and notable limitations worth considering. With the limited availability of HIV vaccines, contribution, need and equality-based sharing approaches ought to be considered. Thus sharing HIV vaccines based on one’s contribution stresses the need to reciprocate benefits to HIV clinical trial stakeholders especially host communities and participants. Need-based sharing pays attention to at-risk and vulnerable populations. That is, access should be prioritized to those who are likely to get the disease and/or spread it regardless of their contributions to HIV vaccine trials. Equality-based sharing ought to be considered for all others to overcome vaccine discrimination in the community. However, rewarding contribution might dovetail with need, assuming that trials enroll somewhat high-risk populations. With that countries in the global south including Tanzania ought to be prioritized to access HIV vaccines when available.

## Data Availability

The datasets used and/or analyzed during the current study are available from the corresponding author upon reasonable request.

## Abbreviations

CABs: Community Advisory Boards
CAG: Community Advisory Group
FGDs: Focus group discussions
IDI: In-depth interviews
HIV: Human immunodeficiency virus
